# When Cryptogenic Is Not Cryptogenic: Carotid Web as a Cause of Recurrent Embolic Stroke

**DOI:** 10.7759/cureus.115138

**Published:** 2026-08-25

**Authors:** Almurtadha Tawfeeq, Nahla Hamza

**Affiliations:** 1 Stroke Medicine, Manchester University NHS Foundation Trust/Trafford General Hospital, Manchester, GBR

**Keywords:** carotid web, cryptogenic strokes, embolic stroke of undetermined source, recurrent ischemic stroke, young adult ischemic stroke

## Abstract

A carotid web is an under-recognized vascular abnormality increasingly identified as a cause of embolic ischemic stroke in younger patients without conventional vascular risk factors. Delayed diagnosis may lead to recurrent cerebrovascular events despite otherwise unrevealing investigations.

We report the case of a 35-year-old woman who experienced three ischemic strokes over a three-year period. Initial investigations, including carotid Doppler ultrasonography, cardiac evaluation, CT angiography, and a young stroke laboratory screen, did not identify a clear etiology. Ongoing clinical suspicion in the setting of recurrent embolic events prompted further vascular reassessment. A subsequent MRI of the neck with magnetic resonance angiography demonstrated an abnormality of the proximal left internal carotid artery suggestive of a carotid web, and hence, repeated CT angiography confirmed the diagnosis. The patient underwent carotid endarterectomy, with operative confirmation of the diagnosis.

This case highlights carotid webs as an important and potentially treatable cause of recurrent embolic strokes. Increased awareness and careful review of vascular imaging may facilitate earlier diagnosis and prevention of further cerebrovascular events.

## Introduction

A carotid web is a shelf-like intraluminal projection arising most commonly from the posterior wall of the carotid bulb or proximal internal carotid artery. Histologically, it is considered an intimal variant of fibromuscular dysplasia. Although uncommon, it has emerged as an important cause of otherwise unexplained ischemic stroke, particularly in younger patients with few traditional vascular risk factors [1, 2].

A recent systematic review and meta-analysis estimated the prevalence of carotid webs at approximately 9.6% among patients with embolic strokes of undetermined source, with a disproportionately higher representation in younger cohorts, women, and individuals of African descent [3]. The lesion has been associated with recurrent ipsilateral ischemic events and is thought to act as a nidus for thromboembolism. Recognition remains challenging, as carotid webs may be subtle on routine imaging, misinterpreted as minimal atherosclerotic plaque, or overlooked entirely. Because the risk of recurrence may be clinically significant, early diagnosis carries important therapeutic implications [2,4].

We present a case of recurrent embolic stroke in a young woman in whom repeated routine investigations were unrevealing until targeted vascular reassessment identified a carotid web.

## Case presentation

A 35-year-old woman with no significant vascular risk factors presented with recurrent neurological events over a period of several years. She initially presented with a headache and a concern for subarachnoid hemorrhage. A CT of the head demonstrated no acute intracranial abnormality, and she was discharged with reassurance.

After one year and four months, during pregnancy, she presented again with a right-sided headache and a concern for cerebral venous sinus thrombosis. Magnetic resonance venography showed no evidence of venous sinus thrombosis. Imaging demonstrated a small focus of T2 hypointensity within the right parietal deep white matter, considered consistent with a possible early cavernoma associated with a developmental venous anomaly. Conservative management was subsequently advised by the neurosurgical team.

Seven months later, she presented again with a worsening headache and no focal neurological symptoms or signs. MRI of the brain demonstrated stable appearances of the right parietal periventricular cavernoma with no evidence of recent hemorrhage, together with a patchy area of acute cortical infarction in the left frontal lobe. She was, therefore, referred by the neurosurgical team to the stroke team.

Ten months later, she continued to experience recurrent headaches and presented to the hospital again. Repeat outpatient MRI of the brain showed essentially unchanged appearances of the supratentorial and infratentorial parenchyma compared with the previous study, with no diffusion abnormality and no acute intracranial pathology or structural cause identified for her symptoms. Following review in the stroke clinic, her symptoms were felt to be most consistent with migraine, and a referral to the headache clinic was planned. Transthoracic echocardiography showed no structural heart abnormality, and ambulatory cardiac rhythm monitoring showed normal sinus rhythm; she was discharged from the clinic.

Three months later, she presented with transient expressive dysphasia and no other focal neurological signs or symptoms. CT of the head showed no acute intracranial abnormality, and she was discharged with an impression of migraine and functional neurological disorder.

Eight months later, she re-presented with expressive dysphasia and was admitted to the hospital. MRI of the brain showed multifocal acute and subacute infarcts within the left middle cerebral artery territory, in keeping with a thromboembolic process. Carotid Doppler ultrasonography showed less than 30% stenosis of the internal and external carotid arteries, with no plaque, intimal dissection, or other abnormality. CT angiography demonstrated normal contrast opacification of the bilateral middle cerebral arteries and circle of Willis, no filling defects to suggest acute thrombus, patent intracranial venous sinuses, and unremarkable extracranial soft tissues and calvarium. She was discharged on dual antiplatelet therapy with outpatient referral back to stroke medicine.

Further outpatient investigation by the stroke team included repeat cardiac assessment with bubble echocardiography, which did not demonstrate a right-to-left shunt, and a repeat five-day Holter monitor, which again showed sinus rhythm. A young stroke laboratory screen, including a thrombophilia screen, anticardiolipin antibodies, beta-2 glycoprotein I antibodies, antinuclear antibodies, and rheumatoid factor, was negative, with the exception of lupus anticoagulant. She was reviewed by the hematology team, who considered this finding to be of uncertain clinical significance given that the remaining screen was negative, and she was discharged.

Eight months later, she presented with right arm weakness and sensory disturbance. CT of the head demonstrated further ischemic change within the left middle cerebral artery territory, and she was again referred to the stroke team.

**Figure 1:**
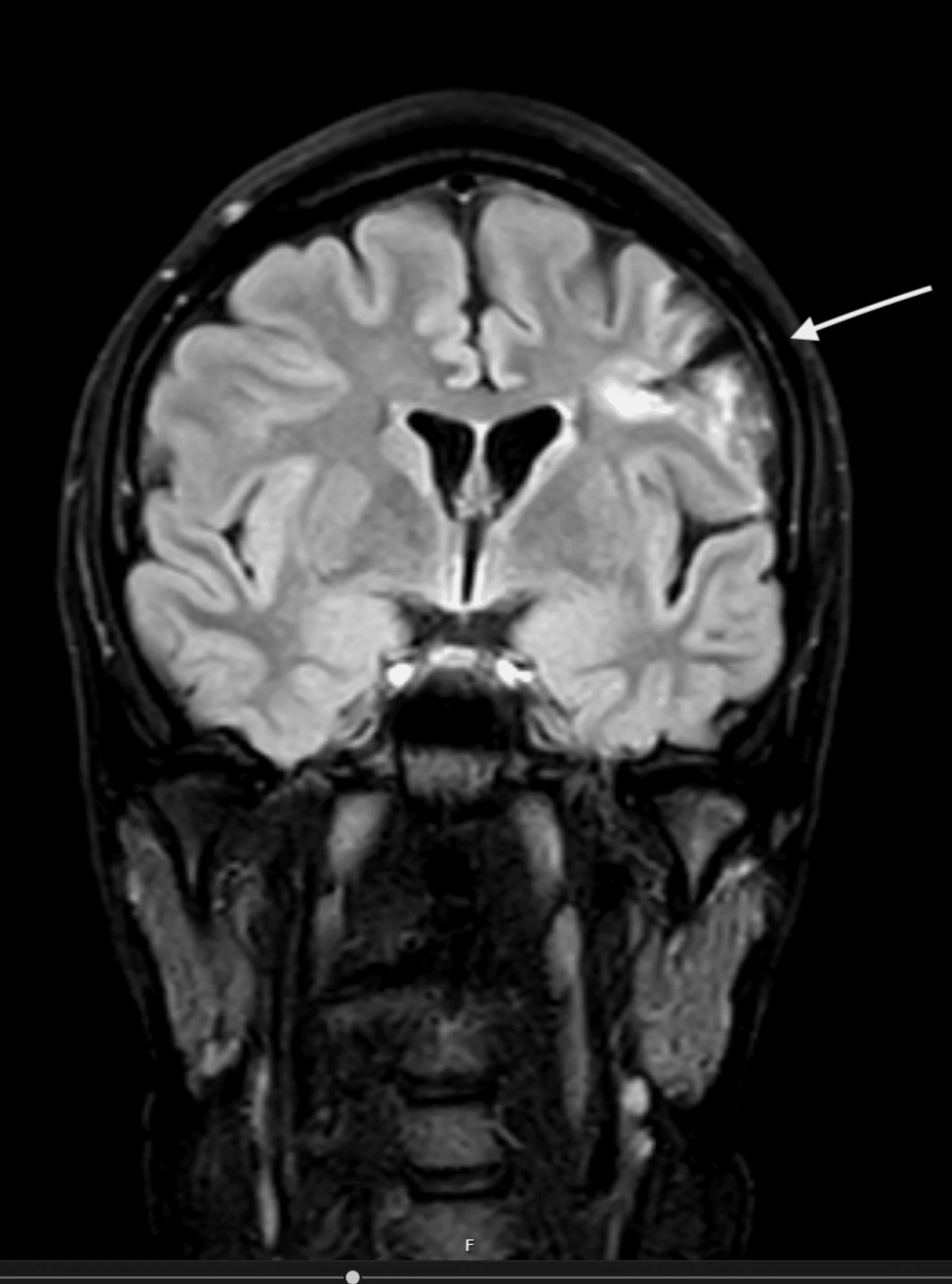
Coronal fluid-attenuated inversion recovery (FLAIR) MRI of the brain demonstrating cortical and subcortical hyperintensity in the left middle cerebral artery territory (arrow), consistent with an established infarction.

**Figure 2:**
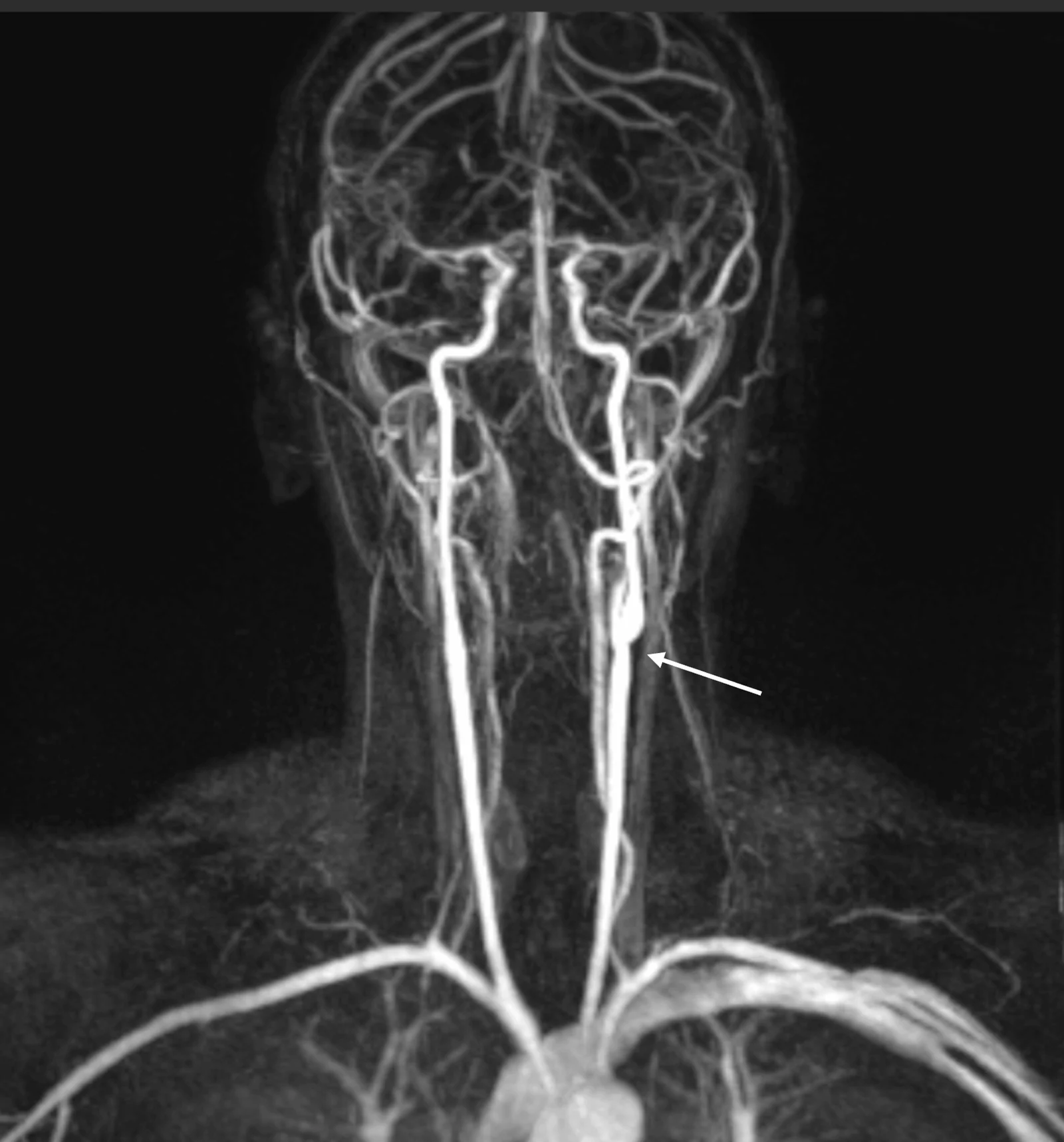
An magnetic resonance angiogram of the neck showing an abnormality at the left internal carotid artery (arrow). The proximal internal carotid artery looks dilated with focal narrowing at the internal carotid artery origin.

**Figure 3:**
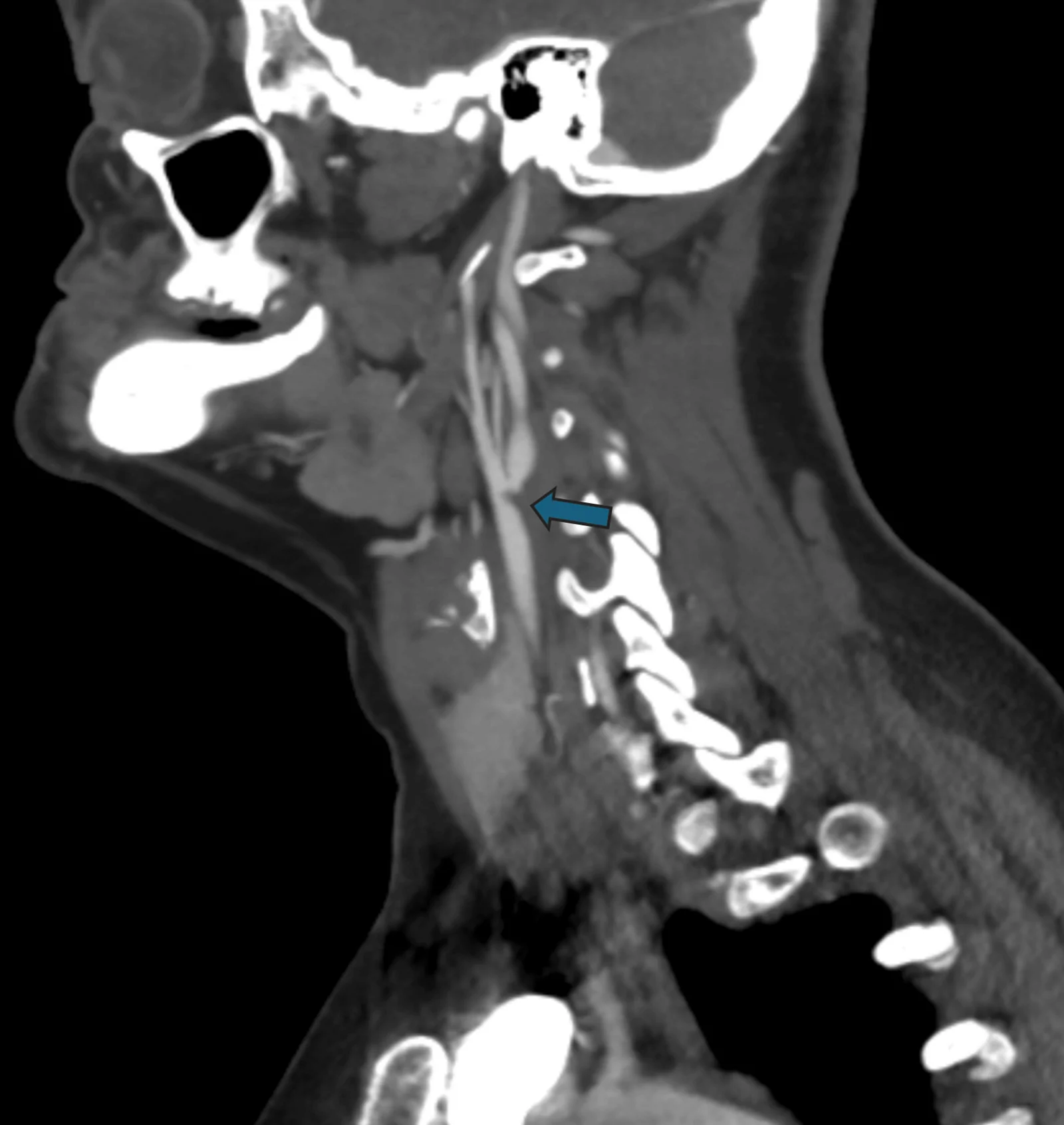
CT angiography of the neck demonstrating a smooth shelf-like hypo-density at the posterior aspect of the internal carotid artery origin (arrowed) causing a mild stenosis (North American Symptomatic Carotid Endarterectomy Trial (NASCET) 31%) with associated post-stenotic dilatation, which is in keeping with a carotid web.

Given this recurrent embolic pattern in a young patient, further vascular reassessment was pursued. Four months later, MRI of the head confirmed an established left frontal lobe infarct (Figure 1), and MRI of the neck with magnetic resonance angiography (Figure 2) demonstrated a focal abnormality involving the proximal left internal carotid artery just beyond the bifurcation, highly suspicious for a carotid web. Repeat CT angiography performed two months later (Figure 3) showed mild stenosis of the left internal carotid artery origin with post-stenotic dilatation, confirming the diagnosis.

The case was reviewed at a multidisciplinary vascular meeting, and the patient was referred for operative management. She subsequently underwent left carotid endarterectomy five months later, with intraoperative confirmation of a carotid web.

A summary timeline of presentations, investigations, and outcomes is presented in Table 1.

**Table 1:** A timeline of clinical presentations, investigations and outcomes. TTE: transthoracic echocardiogram; MCA: middle cerebral artery; CTA: CT angiography; ICA: internal carotid artery

Timepoint	Presentation	Key Investigations & Findings	Management/Outcome
Day 0	Headache; concern for subarachnoid hemorrhage	CT of the head showed no acute intracranial abnormality.	Discharged with reassurance
+1 year 4 months (pregnancy)	Right-sided headache; concern for cerebral venous sinus thrombosis	Magnetic resonance venography done; no venous sinus thrombosis was noted. Small right parietal T2 hyperintensity, ?early cavernoma with developmental venous anomaly	Conservative management (neurosurgery)
+7 months	Worsening headache, no focal signs	MRI of the brain: stable cavernoma; new patchy acute cortical infarct, left frontal lobe	Referred to stroke team
+10 months	Recurrent headaches	Repeat MRI of the brain unchanged, no diffusion restriction; TTE and ambulatory cardiac monitoring normal	Diagnosed as migraine; referred to headache clinic; discharged from stroke clinic
+3 months	Transient expressive dysphasia, no other focal signs	CT of the head: no acute intracranial abnormality	Discharged; migraine/functional neurological disorder
+8 months	Expressive dysphasia, admitted	MRI of the brain: multifocal acute/subacute infarcts, left MCA territory. Carotid Doppler: <30% stenosis, no plaque/dissection. CTA of the head and neck: unremarkable	Dual antiplatelet therapy; outpatient referral to stroke medicine
Interim outpatient work-up	—	Bubble echocardiography: no right-to-left shunt. Five-day Holter: sinus rhythm. Young stroke screen negative except lupus anticoagulant (uncertain significance)	Reviewed by hematology; discharged
+8 months	Right arm weakness and sensory disturbance	CT of the head: further ischaemic change, left MCA territory	Re-referred to stroke team
+4 months	Diagnostic reassessment	MRI of the head: established left frontal infarct. MRI of the neck with MRA: focal abnormality, proximal left ICA, suspicious for carotid web	Further vascular reassessment pursued
+2 months	—	Repeat CTA: mild stenosis of left ICA origin with post-stenotic dilatation	Diagnosis confirmed
+5 months	—	Multidisciplinary vascular meeting review	Left carotid endarterectomy; intraoperative confirmation of carotid web

## Discussion

A carotid web is increasingly recognized as an important cause of cryptogenic and recurrent embolic stroke, particularly in younger patients without significant conventional vascular risk factors. It is considered an intimal variant of fibromuscular dysplasia and typically appears as a thin, shelf-like projection arising from the posterior wall of the carotid bulb or proximal internal carotid artery [1,2].

Observational studies have reported an association between carotid web and ipsilateral anterior circulation ischemic stroke, often in patients without another clear embolic source [1,2,4]. The leading proposed mechanism is thromboembolism related to altered local hemodynamics: the intraluminal projection is thought to create an area of relative flow stasis adjacent to the lesion, predisposing to thrombus formation and subsequent cerebral embolization [5,6].

This case highlights several diagnostic challenges. The patient experienced recurrent embolic events over a prolonged period before the underlying lesion was identified. Initial vascular investigations, including carotid Doppler ultrasonography and CT angiography, failed to identify the lesion. CT angiography is generally regarded as the preferred noninvasive imaging modality for carotid webs, offering high spatial resolution and multiplanar reconstruction that can help distinguish a web from atherosclerotic plaque, dissection, or thrombus [2,5]. Despite this, carotid webs remain subtle, shelf-like lesions that are easily overlooked if the carotid bulb is not specifically and deliberately looked for. In patients with persistent clinical suspicion despite unrevealing standard investigations, further targeted vascular imaging, including dedicated multiplanar CT angiography and/or MRI of the neck with magnetic resonance angiography, may help identify the lesion, as demonstrated in this case [2,5].

Digital subtraction angiography (DSA) remains the historical reference standard for carotid webs but is invasive and is not routinely required when CT angiography or MR angiography findings are sufficiently characteristic; in this case, the diagnosis was considered adequately established on noninvasive imaging and confirmed intraoperatively, and DSA was therefore not pursued.

Recurrence rates with medical therapy alone may be significant in some reported series, although prospective comparative data remain limited. Both carotid endarterectomy and carotid artery stenting have been described in the literature as secondary prevention strategies in selected symptomatic patients, with existing series suggesting that revascularization is associated with lower rates of recurrent stroke than antiplatelet or anticoagulant therapy alone; however, no randomized comparison exists between endarterectomy and stenting, and treatment selection continues to depend on lesion anatomy, patient factors, and multidisciplinary vascular team assessment rather than a single preferred approach [4,7]. Given the absence of standardized management algorithms, we emphasize that decisions regarding intervention should be individualized following multidisciplinary confirmation of the diagnosis, rather than proceeding to invasive treatment on the basis of imaging alone.

## Conclusions

A carotid web is an under-recognized but potentially treatable cause of recurrent embolic stroke. It should be considered in younger patients with cryptogenic or recurrent anterior circulation ischemic events, particularly when standard investigations (Doppler ultrasonography and CT angiography) are unrevealing, as the lesion is easily missed unless the carotid bulb is specifically assessed; dedicated MRI of the neck with magnetic resonance angiography and a repeated CT angiogram can be valuable next steps.

A carotid web is not a benign diagnosis; once confirmed, prompt definitive treatment with carotid endarterectomy or stenting should be pursued since repeated reliance on conservative management alone allowed further strokes to occur before the underlying lesion was addressed.
